# Spheroid Coculture of Hematopoietic Stem/Progenitor Cells and Monolayer Expanded Mesenchymal Stem/Stromal Cells in Polydimethylsiloxane Microwells Modestly Improves *In Vitro* Hematopoietic Stem/Progenitor Cell Expansion

**DOI:** 10.1089/ten.tec.2016.0329

**Published:** 2017-04-01

**Authors:** Kathryn Futrega, Kerry Atkinson, William B. Lott, Michael R. Doran

**Affiliations:** ^1^ Stem Cell Therapies Laboratory, Translational Research Institute, Queensland University of Technology, Brisbane, Australia.; ^2^ Mater Research Institute - University of Queensland, Translational Research Institute, Brisbane, Australia.

**Keywords:** co-culture, cord blood hematopoietic stem cells, engraftment, mesenchymal stem cells, microwell, spheroid culture

## Abstract

While two-dimensional (2D) monolayers of mesenchymal stem/stromal cells (MSCs) have been shown to enhance hematopoietic stem/progenitor cell (HSPC) expansion *in vitro*, expanded cells do not engraft long term in human recipients. This outcome is attributed to the failure of 2D culture to recapitulate the bone marrow (BM) niche signal milieu. Herein, we evaluated the capacity of a novel three-dimensional (3D) coculture system to support HSPC expansion *in vitro*. A high-throughput polydimethylsiloxane (PDMS) microwell platform was used to manufacture thousands of uniform 3D multicellular coculture spheroids. Relative gene expression in 3D spheroid versus 2D adherent BM-derived MSC cultures was characterized and compared with literature reports. We evaluated coculture spheroids, each containing 25–400 MSCs and 10 umbilical cord blood (CB)-derived CD34^+^ progenitor cells. At low exogenous cytokine concentrations, 2D and 3D MSC coculture modestly improved overall hematopoietic cell and CD34^+^ cell expansion outcomes. By contrast, a substantial increase in CD34^+^CD38^−^ cell yield was observed in PDMS microwell cultures, regardless of the presence or absence of MSCs. This outcome indicated that CD34^+^CD38^−^ cell culture yield could be increased using the microwell platform alone, even without MSC coculture support. We found that the increase in CD34^+^CD38^−^ cell yield observed in PDMS microwell cultures did not translate to enhanced engraftment in NOD/SCID gamma (NSG) mice or a modification in the relative human hematopoietic lineages established in engrafted mice. In summary, there was no statistical difference in CD34^+^ cell yield from 2D or 3D cocultures, and MSC coculture support provided only modest benefit in either geometry. While the high-throughput 3D microwell platform may provide a useful model system for studying cells in coculture, further optimization will be required to generate HSPC yields suitable for use in clinical applications.

## Introduction

Hematopoietic stem cell (HSC) transplantation is a routine therapy used in the treatment of hematological diseases, including malignancies and immunodeficiencies.^1,2^ Umbilical cord blood (CB) is a suitable alternative transplantable cell population when a matched bone marrow (BM) donor is not available. However, transplantation of the limited number of HSCs that are present in single CB units is associated with delayed engraftment and increased graft failure and mortality.^1^ This has motivated the development of *ex vivo* expansion technologies designed to increase CB HSC numbers.

From a clinical perspective, the previous few years have delivered promising *ex vivo* expansion systems that incorporated bound signal molecules (Notch ligand: >100-fold CD34^+^ expansion^3^), involved coculture with mesenchymal stem/stromal cells (MSCs) (approximately 40-fold CD34^+^ cell expansion^4,5^), or utilized pharmaceutical compounds (e.g., antagonist of the aryl hydrocarbon receptor, StemRegenin 1: approximately 50-fold CD34^+^ expansion^6^). In phase I clinical trials, CB expansion protocols generated sufficient cell numbers to enable accelerated hematopoietic and immune reconstitution in adult patients undergoing CB transplantation.^3–6^ These trials relied on CD34^+^ cell fold expansion as an indicator of HSC-enriched cells in expansion products.^3–6^ Unfortunately, cells from these expanded cell products lacked long-term engraftment potential,^3–5^ making cotransplantation of a second unmanipulated CB unit necessary. It is critical to note that while freshly isolated CD34^+^ cells contain a population of long-term engrafting HSCs, most CD34^+^ cells are lineage-restricted progenitor cells and do not have long-term engraftment potential.^7^ The failure of expanded CD34^+^ populations to engraft for long term suggests that manipulated CD34^+^ cells may not be equivalent to unmanipulated CD34^+^ cells. Because of the limited capacity to distinguish between HSCs and early progenitor cells,^7,8^ these heterogeneous populations are often referred to as, more generally, hematopoietic stem/progenitor cell (HSPCs) and not HSCs.^9^ Overall, clinical experiences with expanded CB products suggest that the large numbers of HSPCs generated through *ex vivo* expansion do not engraft for long term in human recipients.

The difficulty and cost associated with procurement of two or more CB units to provide a manipulated and unmanipulated product for transplantation pose barriers to the commercial and clinical translation of this approach.^1^ Strategies that rely on coculture with MSCs to expand HSPCs require yet another significant investment to manufacture the MSC support cell population. Given that similar, or greater, CD34^+^ cell expansion (50- to 100-fold) can be achieved with immobilized ligands^3^ or pharmaceuticals,^6^ the additional expense of MSC manufacture is only justifiable if the expansion culture could maintain a large population of long-term engrafting HSCs. If this were possible, recipients would not require cotransplantation of a second unmanipulated unit of CB, and this saving could be used to offset the cost of MSC manufacture.

In the adult BM niche, HSCs have been shown to colocalize with MSCs, which express HSC maintenance factors (the role of MSCs in the BM niche is reviewed by Mendelson and Frenette^10^ and Bianco^11^). The HSPC-MSC coculture system that was evaluated clinically utilized a two-dimensional (2D) monolayer of MSCs to support the expansion of CB-derived CD34^+^ cells seeded on top of the monolayer.^4,5^ These expanded cells did not engraft for long term in human recipients.^5^ The failure of MSC cocultures to support the maintenance of long-term engrafting HSCs suggested that these cultures did not adequately recapitulate the microenvironment of the BM niche. Despite failure to support *ex vivo* HSC self-renewal, the use of MSCs as a support cell population in coculture is a rational starting point due to their biological association in the BM niche. A number of groups have begun to develop strategies to improve HSPC-MSC coculture outcomes. These include the use of MSCs enriched for subpopulations known to exhibit more potent HSC-supportive properties,^12,13^ using scaffolds to allow formation of three-dimensional (3D) tissues and enhanced cell–cell interactions,^14^ and through the use of 3D MSC spheroids.^12,15^ An increasing number of studies suggest that the HSC-supportive properties of both human^12,15^ and murine^16,17^ MSCs are enhanced when these cells are cultured as spheroids. In these previous studies, MSC spheroid sizes were large and/or heterogeneous. We reasoned that the development of a high-throughput uniform spheroid coculture model system would allow us to optimize *in vitro* HSPC coculture expansion and reveal whether true benefits could be achieved using such a platform.

Building on the previous work discussed above, we hypothesized that 3D spheroid coculture of human CB-derived CD34^+^ cells with BM-derived MSCs might enhance the supportive properties of MSCs and improve cell–cell interactions between the MSCs and CD34^+^ cells. Herein, we tested this hypothesis through the development and evaluation of a high-throughput polydimethylsiloxane (PDMS) microwell platform used to manufacture hundreds of uniform, 3D, multicellular coculture spheroids. The use of a high-throughput platform to assemble uniform human MSC-CD34^+^ cocultures has not been reported, and we reasoned that such a platform would enable reliable and reproducible evaluation of the spheroid coculture approach. Coculture spheroids were manufactured to contain various numbers of MSCs, ranging from approximately 25—to 400 MSCs each and 10 CB-derived CD34^+^ cells each. Three-dimensional MSC spheroid cultures were assessed for relative gene expression using a microarray and their ability to support the expansion of CD34^+^ cells relative to 2D MSC cocultures.

## Materials and Methods

### MSC isolation

MSCs were isolated from 20 mL BM aspirates collected from the iliac crest of healthy, consenting adult donors. The Mater Health Services Human Research Ethics Committee and the Queensland University of Technology Human Ethics Committee approved aspirate collection (Ethics No. 1541A). MSCs were isolated as described previously.^18^ MSCs were expanded in medium containing low-glucose Dulbecco's modified Eagle's medium (DMEM; Life Technologies), 10% fetal bovine serum (FBS; Life Technologies), 10 ng/mL fibroblast growth factor-1 (FGF-1; PeproTech), and 100 U/mL penicillin/streptomycin (PenStrep; Life Technologies) in a 2% O_2_ and 5% CO_2_ atmosphere at 37°C. MSCs were used up to passage 4 for experiments.

### CD34^+^ isolation from CB

CB was collected at the Mater Hospital in Brisbane from full-term births after obtaining informed consent from mothers. Ethics approval was granted by the Mater Health Services Human Research Ethics Committee and the Queensland University of Technology Human Ethics Committee (Ethics No. 1100000210). CD34^+^ cells were isolated from CB within 24 h of collection using the human CD34 MicroBead Kit (Miltenyi) as previously described.^19^

### Fabrication and surface modification of microwell plates (made in-house) for 3D spheroids

In-house fabricated microwell plates were used to manufacture multicellular spheroids in all experiments, except for the MSC gene expression studies, where AggreWell six-well plates (StemCell Technologies) were used instead. The in-house fabricated microwell platform was manufactured from PDMS (Dow Corning Sylgard 184). As previously described, a microwell mold was used to generate sheets of PDMS with the microwell pattern, and a punch was used to prepare inserts that fitted snuggly into 48-well culture plates.^20^ To sterilize the microwell-containing plates, 70% ethanol was added to each well and centrifuged at maximum speed to remove any air bubbles. The plates were then completely submerged in 70% ethanol for 1 h. The wells were rinsed four times with sterile water for at least 1 h of soaking per rinse in a sterile flow cabinet. For storage, plates were dried first in an oven overnight. To prevent cell attachment to the PDMS microwells, the wells were coated with 5% Pluronic F127 (Sigma) solution for 5 min and rinsed three times with phosphate-buffered saline (PBS) before cell seeding.^18^

### MSC characterization

MSCs were characterized using flow cytometry (LSRII; BD Biosciences) for surface expression of CD44, CD73, CD90, CD105, and CD146 and the absence of hematopoietic markers, including CD45, CD34, and HLA-DR. All antibodies and isotype controls were from Miltenyi, and cells were stained as per the manufacturer's instructions.

Trilineage mesodermal differentiation down the osteogenic, adipogenic, and chondrogenic lineages was assessed in 3D MSC spheroid cultures. MSCs were seeded with induction media at 60 × 10^3^ cells per well (100 cells per spheroid) in 48-well plates containing PDMS microwell inserts and centrifuged at 100 *g* for 3 min to force cell aggregation; this resulted in spheroids of ∼100 MSCs each. Osteogenic induction media contained high-glucose (HG)-DMEM supplemented with 10% FBS, PenStrep, 50 μM ascorbic acid 2-phosphate (Sigma), 10 mM β-glycerol phosphate (Sigma), and 10^−7^ M dexamethasone (Sigma). Adipogenic induction media contained HG-DMEM supplemented with 10% FBS, PenStrep, 10 μg/mL insulin (Sigma), 200 μM indomethacin (Life Technologies), 500 μM 3-isobutyl-1-methyl xanthine (Sigma), and 10^−7^ M dexamethasone. Chondrogenic induction media contained HG-DMEM supplemented with PenStrep, 1 × insulin-transferrin-selenium-ethanolamine (ITS-X; Life Technologies), 40 μg/mL l-proline (Sigma), 110 μg/mL sodium pyruvate (Life Technologies), 200 μM ascorbic acid 2-phosphate, 10 ng/mL recombinant human transforming growth factor-β1 (TGF-β1; PeproTech), and 10^−7^ M dexamethasone. Adipogenic and osteogenic induction cultures were incubated in a 20% O_2_ and 5% CO_2_ atmosphere at 37°C as previously described.^21^ Chondrogenic induction cultures were incubated in a 2% O_2_ and 5% CO_2_ atmosphere at 37°C as previously described.^18,22^ Induction was carried out for 21 days, with medium exchange every 3–4 days.

Induced MSC spheroids were fixed with 4% paraformaldehyde (Sigma) for 30 min. To assess osteogenic induction, hydroxyapatite was stained for using the OsteoImage Mineralization Assay (Lonza) as per the manufacturer's instructions. To assess adipogenic induction, lipids were stained with Oil Red O (Sigma) stain and 4′,6-diamidino-2-phenylindole (DAPI; Life Technologies) was used to visualize cell nuclei. Osteogenic and adipogenic induced spheroids were imaged using a Leica TCS SP5 confocal microscope. To assess chondrogenic induction, spheroids were cryosectioned and stained with Alcian blue stain (Sigma) for the presence of glycosaminoglycans (GAG).

### MSC 2D and 3D gene expression analysis

Parallel 2D adherent and 3D spheroid MSC cultures were prepared from a single expanded MSC donor (55-year-old male) at passage 3. For 2D MSC cultures, six-well plates were first precoated with fibronectin (BD Biosciences) at 10 μg/mL for 1 h to ensure MSC attachment to the well surface. Three-dimensional cultures were established in AggreWell 400Ex six-well plates (StemCell Technologies). Six-well AggreWell plates were used in this experiment due to their larger size, which was required to obtain sufficient quantities of total RNA. AggreWell plates were rinsed with 5% Pluronic F127 to prevent cell attachment to the microwells.^18^ All plates were seeded with 5 × 10^5^ MSCs in 4 mL of X-Vivo 15 medium (Lonza), yielding ∼100 cells per spheroid. AggreWell plates were centrifuged at 100 *g* for 3 min to force cell aggregation. Following 3 and 6 days of culture, 2D and 3D MSC cultures were collected for total RNA extraction. At each time point, four replicate wells from 2D cultures and four replicate wells from 3D cultures were washed with PBS and total RNA was isolated for each well using the RNeasy Mini Kit (Qiagen) with on-column DNase (Qiagen) digestion step, as per the manufacturer's instructions. RNA quantity and quality were determined using an Agilent 2100 Bioanalyzer and RNA 6000 Nano Kit (both from Agilent Technologies), as per the manufacturer's instruction.

Microarray gene expression analysis was performed at the Australian Genome Research Facility (AGRF, Melbourne, Australia) using the Illumina HumanHT-12 Expression BeadChip Kit (Illumina), as per the manufacturer's instructions, and as reported previously.^23^ Using GenomeStudio, version 1.9.0, scanner software, the chip signal arrays were converted into text files for analysis. Probe sample profiles and control profiles were uploaded into the statistical programming language R (version 3.1.2).^24^ Bioconductor^25^ packages lumi^26^ and arrayQualityMetrics^27^ software packages were used for quality control. All measured samples passed quality control. Data were further processed using the Bioconductor package limma.^28,29^ Output values were treated with normexp background correction using negative controls and quantile normalization and then log2 transformed. Differentially expressed genes were compared between MSCs grown in 3D spheroid cultures and 2D adherent cultures on days 3 and 6. Gene ontology enrichment analysis was performed on the list of differentially expressed probes in Partek (Genomic Suite software, version 6.6). The statistical significance of over-represented genes was calculated using Fisher's exact test. An enrichment score of >3 (*p* value <0.05) was considered to be significantly overexpressed in the annotated functional category. Differentially expressed genes in the current study were contrasted against those deemed particularly interesting in similar published studies.^30,31^

### Preparation of CD34^+^ and MSC cocultures

**FIG. 1.**
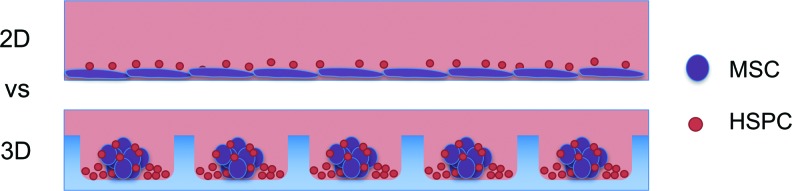
Schematic of HSPC/MSC cocultures in 2D and 3D. Two-dimensional cocultures (*top panel*) were established on adherent fibronectin-coated tissue culture plastic surfaces. Fibronectin facilitated MSC adherence to the 2D tissue culture plastic in the serum-free culture medium. Three-dimensional cocultures (*bottom panel*) were established in nonadherent Pluronic F127-coated PDMS microwells. Pluronic F127 coating blocked cell attachment to the PDMS and preferentially promoted cell aggregation.^14^ Microwell dimensions: 360 × 360 μm wide and 180 μm deep with ∼600 microwells/cm^2^. 2D, two-dimensional; 3D, three-dimensional; HSPC, hematopoietic stem/progenitor cell; MSCs, mesenchymal stem/stromal cells; PDMS, polydimethylsiloxane. Color images available online at www.liebertpub.com/tec

A schematic representation of 2D and 3D cocultures is shown in Figure 1. Plates used for 2D culture were precoated with fibronectin to promote MSC attachment to the tissue culture plastic (TCP) well surface, while 3D microwell surfaces were precoated with 5% Pluronic F127 to prevent MSC attachment to the microwell surface, as described above.^18^ Cells were cultured in 0.5 mL of serum-free X-Vivo 15 media supplemented with human recombinant cytokines at specified concentrations. Each well of a 48-well plate was seeded with a starting CD34^+^ cell density of 6 × 10^3^ per well or 1 cm^2^. At this seeding density, ∼10 CD34^+^ cells would be contained in a single microwell in the 3D culture platform. Similarly, at 60 × 10^3^ MSCs per well of a 48-well plate, ∼100 MSCs would be seeded in a single microwell. Microwell plates were centrifuged at 100 *g* for 3 min to aggregate cells. Cells were cultured in a 2% O_2_ and 5% CO_2_ atmosphere at 37°C for 7 days. To determine the capacity of MSCs in 2D or 3D to improve CD34^+^ cell expansion, low exogenous cytokine conditions (10 ng/mL SCF) were used. Control cultures containing more common high cytokine conditions (100 ng/mL of SCF, 100 ng/mL TPO peptide^32^ [referred to as TPO hereafter; Auspep Pty] and 50 ng/mL Flt-3 ligand) were carried out in parallel. The high cytokine condition utilized early acting cytokines^33^ and is similar to that used in a recent 3D MSC coculture study.^12^ MSC seeding density was varied in low cytokine cultures to determine if increasing levels of MSCs correlated with improvement in CD34^+^ expansion. Four replicate wells were evaluated for each culture condition, and five replicate experiments were performed using different MSC and CB donors.

### Flow cytometry and confocal analysis of HSPC expansion

Flow cytometry analysis of trypsin-dissociated cultures was performed using antibodies for CD45-FITC, CD34-APC, and CD38-PE and corresponding isotype controls IgG2a-FITC, IgG2a-APC, and IgG2a-PE, all from Miltenyi and used as per the manufacturer's instructions. Cells were enumerated using fluorospheres (Beckman Coulter). Hematopoietic cells were assessed based on positive CD45 surface expression and progenitor cells were quantified based on positive CD34 surface expression and lack of CD38 expression. For gating strategy, see Supplementary Figures S1 and S2 (Supplementary Data are available online at www.liebertpub.com/tec).

For 3D imaging of coculture spheroids, cells were prepared by first labeling MSCs with Cell Tracker^™^ Red CMTPX (Molecular Probes) and CD34^+^ cells with Green CellTrace^™^ CFSE (Molecular Probes), as previously described.^19^ Following 7 days of coculture, spheroids were fixed, washed of detached cells using a cell strainer, and imaged on a Leica TCS SP5 confocal microscope.

### Transplantation of CD34^+^ cells expanded on 2D TCP or 3D PDMS microwells

To determine if the PDMS 3D microwell insert modified engraftment potential, expanded CD34^+^ cells were transplanted into sublethally irradiated adult NOD/SCID gamma (NSG) male mice. The University of Queensland (UQ) and the Queensland University of Technology (QUT) Animal Ethics Committees authorized these animal procedures (Ethics No. 1300000644). NSG mice were purchased from the Jackson Laboratory^34^ and bred in the Animal Facility at the Translational Research Institute (TRI, Brisbane, Australia).

Expansion cultures were initiated from 50 × 10^3^ CD34^+^ cells per well in six-well plate control wells (TCP with no PDMS) or with cured PDMS microwell inserts in the bottom of six-well plates. For the engraftment assays, two different CB donors were used in two independent animal experiments. Following 7 days of culture, expanded cells were collected from each well and resuspended in 100 μL of fresh X-Vivo 15 media. Twenty-four hours before transplant, mice were irradiated with 250 cGy using a Gamma Cell 40 Cesium source. On the day of transplant, mice were anesthetized by isoflurane inhalation and cells injected into the retro-orbital sinus. In each experiment, eight mice were transplanted with TCP- or PDMS-expanded cell product, each from a single original well.

Human (h) cell engraftment in the NSG mice was assessed at 8 weeks. Relative human engraftment in the BM, spleen, and peripheral blood was quantified using flow cytometry analysis performed on an LSRII (BD Biosciences), and data were analyzed using FlowJo software (Tree Star) as previously described.^19^ Positive human (h) engraftment was defined as more than 1% hCD45^+^ in one of the mouse tissues analyzed. Nonengrafted mice were excluded from subsequent lineage analysis.

### Statistical analyses

Statistical analyses for microarray gene expression studies were performed by the AGRF and are described in the relevant sections. This analysis was only completed for a single MSC donor. Coculture data from five replicate experiments are shown in Figure 6. For these data sets, a common MSC donor was used for CB populations 1–3, while two independent MSC donors were used to support CB populations 4 and 5. All five CB units were derived from unique donors. Data for each expansion coculture are presented as mean ± standard deviation for four replicate wells. Statistical significance of data was evaluated using two-way analysis of variance (ANOVA), or *t*-tests where specified, in Prism software, version 5.0 (GraphPad). The *p* values obtained in each comparison are represented by asterisks in graphs as follows: *p < 0.05, **p < 0.01, ***p < 0.001, and ****p < 0.0001.

## Results

### Manufacture of 3D MSC spheroid cultures and characterization

**FIG. 2.**
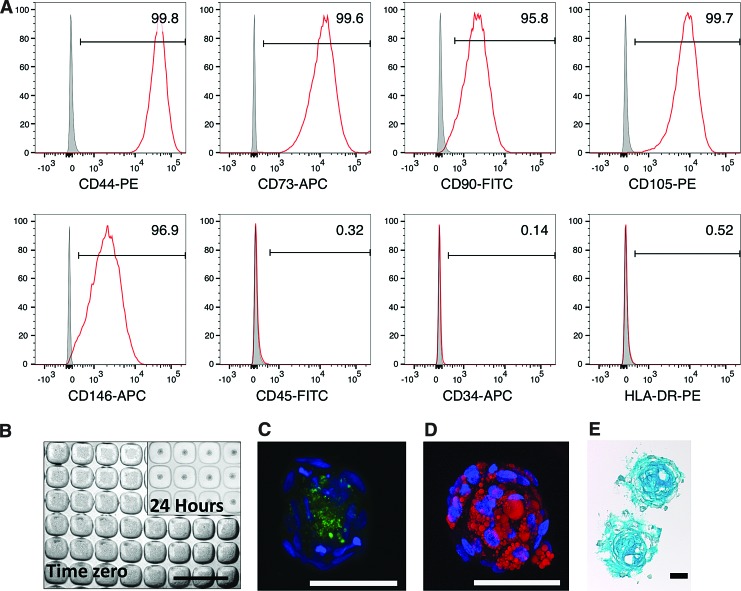
MSC characterization. **(A)** Flow cytometry characterization of MSC surface markers (CD44, CD73, CD90, CD105, and CD146) and absence of hematopoietic markers (CD45, CD34, and HLA-DR). **(B)** MSC spheroid manufacture in the PDMS microwell platform. Each microwell was 360 × 360 μm by 180 μm deep. Seeding of cells into microwells resulted in ∼100 cells/microwell. Within 24 h, cells aggregated into spheroids of ∼100 μm in diameter. Scale bar = 1 mm. **(C)** Confocal images of MSC spheroids differentiated down to the osteogenic lineage (hydroxyapatite, *green*). **(D)** Adipogenic lineage (oil droplets, *red*) (nuclei stained with DAPI, *blue*). **(E)** Cryosection from MSC spheroids differentiated down the chondrogenic lineage (GAG, *blue*). **(C–E)** Scale bar = 50 μm. DAPI, 4′,6-diamidino-2-phenylindole; GAG, glycosaminoglycans; PDMS, polydimethylsiloxane. Color images available online at www.liebertpub.com/tec

Based on flow cytometry analysis, expanded MSCs were >95% positive for the MSC-associated markers, CD44, CD73, CD105, CD90, and CD146, and <5% positive for CD45, CD34, and HLA-DR (Fig. 2A). The use of a microwell platform, fabricated in-house, enabled the manufacture of hundreds of uniformly sized MSC spheroids. Figure 2B shows an image of the microwell platform seeded with ∼100 MSCs per microwell and an inset image of MSCs that had aggregated into spheroids after 24 h. At this seeding density, MSC spheroids were ∼100 μm in diameter.

MSCs differentiated in 3D spheroid cultures toward osteogenic, adipogenic, and chondrogenic lineages (Fig. 2C–E). Our team has previously characterized both osteogenic^21^ and chondrogenic^18,22^ differentiation in similar sized MSC spheroids. Punctate hydroxyapatite nodule formation (green) was observed toward the core of the spheroid (Fig. 2C). This was consistent with previous reports showing dense mineralization located within the core of osteogenically induced MSC spheroids.^35^ Adipogenic induction of MSC spheroids resulted in the formation of lipid droplets (red), which were distributed throughout the spheroids (Fig. 2D). Chondrogenically induced MSC spheroids yielded uniform matrix (GAG, blue) staining (Fig. 2E). Overall, our results mirror previous studies,^21,22,35,36^ demonstrating that MSC trilineage mesodermal differentiation is inducible in 3D spheroids using a microwell platform.

### MSC 2D and 3D gene expression analysis using microarrays

Differentially expressed genes were evaluated between the 3D MSC spheroid cultures and 2D adherent MSC cultures on days 3 and 6 (Table 1). A list of differentially expressed genes is available in Supplementary Table S1. We identified more than 140 genes that were significantly upregulated, more than twofold, and more than 200 genes that were downregulated, more than twofold, at both time points. Table 1 shows the top 20 most upregulated genes and the top 20 most downregulated genes.

**Table 1.** Top 20 Most Upregulated and Top 20 Most Downregulated Genes in Mesenchymal Stem/Stromal Cells Cultured in Three-Dimensional Spheroids Compared with Two-Dimensional Adherent Cultures at Days 3 and 6

*Day 3*			*Day 6*		
*Array ID*	*Gene*	*Fold change*	*Array ID*	*Gene*	*Fold change*
Upregulated genes in MSC spheroid culture					
3840154	SPP1	61.9	5900564	SLC16A6	39.6
7150634	APOD	27.4	3840154	SPP1	33.8
5900564	SLC16A6	22.3	7150634	APOD	26.5
2750367	PMAIP1	19.2	3290368	IGFBP1	20.9
5260095	GJB2	18.9	7200609	PCSK1	17.4
4280577	BMP2	14.8	3060735	IL1RN	15.9
2030598	CNIH3	12.9	5260095	GJB2	14.9
5340136	GALNT9	12.8	6980064	BMP6	13.9
3290368	IGFBP1	12.2	4290201	IL24	12.5
670731	CYTL1	11.2	2750367	PMAIP1	12.5
5260047	FABP5	10.9	7400497	IGFBP1	12.2
6980064	BMP6	10.4	2320598	NDP	12.0
6510608	BTBD11	10.1	5080543	TFPI2	11.9
5900288	LOC728510	10.1	4280577	BMP2	11.7
7510195	TGFB2	10.0	2030598	CNIH3	11.1
650639	NR4A2	9.3	5340136	GALNT9	10.7
990717	LOC642956	8.8	520719	IL1F8	9.2
2060440	MAFB	8.5	5900288	LOC728510	9.1
5080543	TFPI2	8.2	3060008	C2CD4B	9.0
4540475	FABP5 L2	8.2	3840148	NCCRP1	8.9
Downregulated genes in MSC spheroid culture					
1850564	LBP	−28.5	5690687	CTGF	−72.3
3460682	PTX3	−22.6	2360326	TAGLN	−52.8
5690687	CTGF	−19.4	3930605	CYR61	−35.5
6840192	ECM2	−16.8	4070356	FLG	−24.1
1260040	EFEMP1	−16.3	6560112	ANKRD1	−22.3
5960296	CLDN1	−14.9	5960296	CLDN1	−21.1
6560112	ANKRD1	−11.9	6840192	ECM2	−20.9
50446	INHBB	−11.0	2140707	SLPI	−18.8
3830093	LMCD1	−11.0	5050523	KRTAP1-5	−16.9
5810685	THBS1	−11.0	5570102	DKK1	−16.6
2640292	CTGF	−10.4	2570154	MFAP5	−16.5
4070356	FLG	−10.2	3460682	PTX3	−15.5
3930605	CYR61	−10.1	3840458	LEP	−15.1
3310520	MOXD1	−9.0	2190184	PRSS23	−14.7
5270519	ALPK2	−8.6	5270519	ALPK2	−14.6
580441	LOC730833	−8.6	6510170	IFIT3	−13.2
3800095	DEPDC6	−8.1	1580546	LOC728946	−12.8
1450161	C5ORF46	−8.0	3710168	KRT34	−12.8
2140707	SLPI	−7.7	6330079	EGFL6	−12.5
5420689	ECM2	−7.4	580441	LOC730833	−12.4

Values are fold changes for 3D MSC spheroid cultures compared with 2D adherent MSC cultures. Positive values represent upregulated genes and negative values specify downregulated genes.

2D, two-dimensional; 3D, three-dimensional; MSCs, mesenchymal stem/stromal cells.

**FIG. 3.**
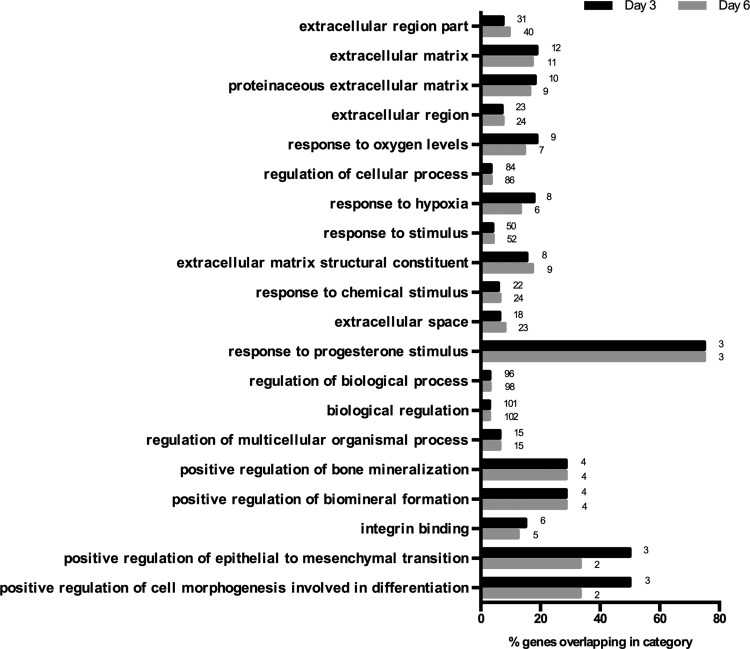
Gene ontology analysis on genes differentially expressed in 3D MSC spheroids compared with 2D adherent MSC cultures. The bars in the graph represent the percentage of genes over-represented in each functional group and the numbers next to each bar represent the number of differentially expressed genes that are found overexpressed in the functional category.

In Table 2, a number of differentially expressed genes were contrasted from our current study and those previously reported by Potapova *et al.*^37^ and those reported to be significantly upregulated by Bartosh *et al.*^31^ Values for fold change in gene expression for previous studies were obtained from the Supplementary Data provided in both of these previous publications. As in our study, Potapova *et al.*^37^ performed gene array analysis of a single MSC donor, Bartosh *et al.*^31^ assessed two MSC donors. Fold changes of only a select set of genes upregulated^31^ have been published; therefore, these values were used as the comparison benchmark. Where a gene was downregulated in Table 2, rather than upregulated, a downward arrow has been used to indicate this direction (↓). The purpose of this comparison was to determine if MSC spheroid culture consistently resulted in similar gene expression changes, and whether these trends would be maintained despite the protocol differences between the three studies (e.g., MSC isolation and culture protocols were different in all three laboratories). Finally, differentially expressed genes from our study that were over-represented in functional gene ontologies are shown in Figure 3.

**Table 2.** Fold Change in Gene Expression for Three-Dimensional Mesenchymal Stem/Stromal Cell Spheroids Compared with Two-Dimensional Adherent Mesenchymal Stem/Stromal Cell Cultures Reported in Previous Studies^12,13^ and in the Current Study

	*Bartosh et al.*^31^			*Current study*	
*Gene name*	*Donor 1, day 3*	*Donor 2, day 3*	*Potapova et al.*^37^*Day 3*	*Day 3*	*Day 6*
Secreted molecules					
IL8	82	34	78.3	NS	2.5
TSG-6	61	40	4.5	1.3 ↓	1.5
IL1B	24	12	3.4	3.0	7.0
BMP2	14	12	32.4	14.8	11.7
CXCL1	12	3	13.5	1.4 ↓	2.2
SPP1	12	5	4.6	61.9	33.8
GDF15	6	6	1.2	1.3	1.4
IL11	10	10	16.2	2.8	3.5
LIF	12	9	2.7	2.5	8.8
SMOC1	8	4	0.44	2.6	NS
IL1A	7	3	6.1	2.9	8.0
IGFBP1	NR	NR	4.2	12.2	20.9
IGFBP5	11	14	1.9	1.7	1.3
C1S	4	3	0.1	1.7 ↓	1.3 ↓
BMP6	8	4	1.1	10.4	13.9
TRAIL	7	6	3.6	1.3	1.3
PTHLH	6	3	3.3	1.4	1.3
NMB	5	3	1.6	NS	NS
APOD	5	3	2.8	27.4	26.5
PLTP	7	4	0.8	NS	1.8
IL24	6	7	1.8	5.2	12.5
IL6	3	3	1.0	1.5 ↓	1.4
STC1	7	10	19.3	1.3 ↓	NS
NAMPT	5	3	NS	1.3	2.3
TGFB2	NR	NR	0.4	10.0	5.3
TGFB3	NR	NR	1.1	6.4	3.9
WNT5A	NR	NR	0.6	2.5	2.6
HGF	NR	NR	0.7	4.4	1.5
CXCL12	NR	NR	NS	4.2 ↓	2.3 ↓
Cell surface receptor					
ITGA2	23	18	4.1	5.8	3.3
EDNRA	15	9	3.5	4.1	6.1
GPR84	13	5	4.1	2.0	3.9
BDKRB2	10	6	1.0	1.8 ↓	3.3
CXCR4	7	5	15.6	2.0	3.1
DPP4	6	4	0.7	0.4	4.0 ↓
CD82	5	4	0.4	NS	1.8
PLA2R1	5	4	0.4	2.1	1.7
PTGDR	7	5	0.9	1.6	1.3
ICAM1	6	5	0.9	1.4 ↓	NS
COLEC12	4	6	0.4	1.3	1.3
C3AR1	5	3	0.2	1.4	1.3
Extracellular matrix molecules					
MMP13	66	37	8.1	1.8	1.9
CHI3 L1	33	36	0.5	1.5	3.3
TFPI2	55	53	5.2	8.2	11.9
MMP3	15	6	3.2	2.2	2.9
MMP1	11	16	94.8	1.4 ↓	1.8
ADAMTS5	7	3	0.5	NS	1.8 ↓
GPC6	4	2	0.4	NS	1.2
LUM	3	3	0.2	2.8 ↓	1.5 ↓
LAMA4	3	3	0.9	1.3 ↓	1.2 ↓
Transcription factors					
NR4A2	12	10	3.8	9.3	8.6
ETV1	11	6	0.5	2.5	1.7
MAFB	9	6	0.9	8.5	4.0
SATB1	6	7	4.5	NS	NS

Values are fold changes for 3D MSC spheroid cultures compared with 2D adherent MSC cultures. Table modified from Supplementary Data in Bartosh *et al*.^31^

Values represent upregulated genes, unless specified with a downward arrow ↓.

NR, not reported; NS, not significant.

### Two-dimensional and 3D coculture characterization

**FIG. 4.**
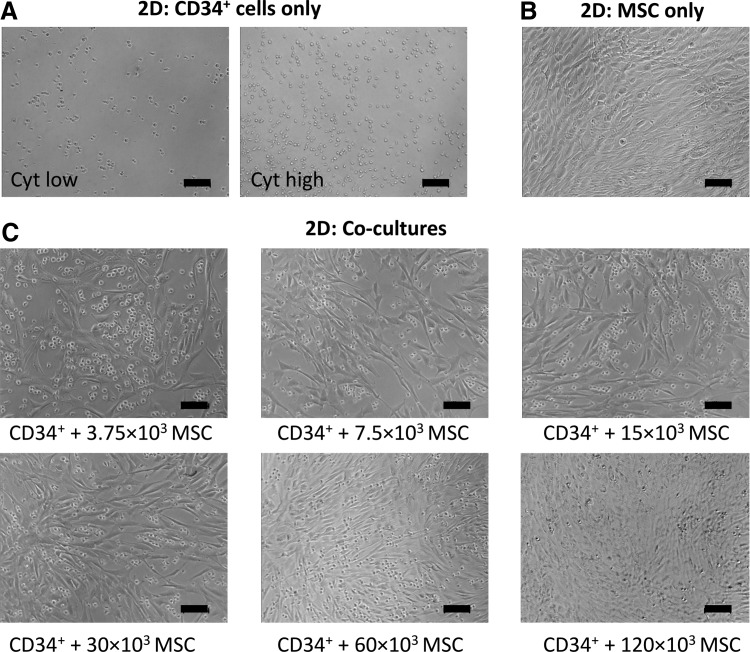
Phase-contrast images of 2D cultures after 7 days of expansion. Cultures were prepared containing either 6 × 10^3^ CD34^+^ cells alone (10 per spheroid) in low cytokine conditions (*left panel*) or high cytokine conditions (*right panel*) **(A)**; 60 × 10^3^ MSCs only **(B)**; or 6 × 10^3^ CD34^+^ cells and variable numbers of MSCs, as specified *below* images, in low cytokine conditions **(C)**. Scale bar = 100 μm.

**FIG. 5.**
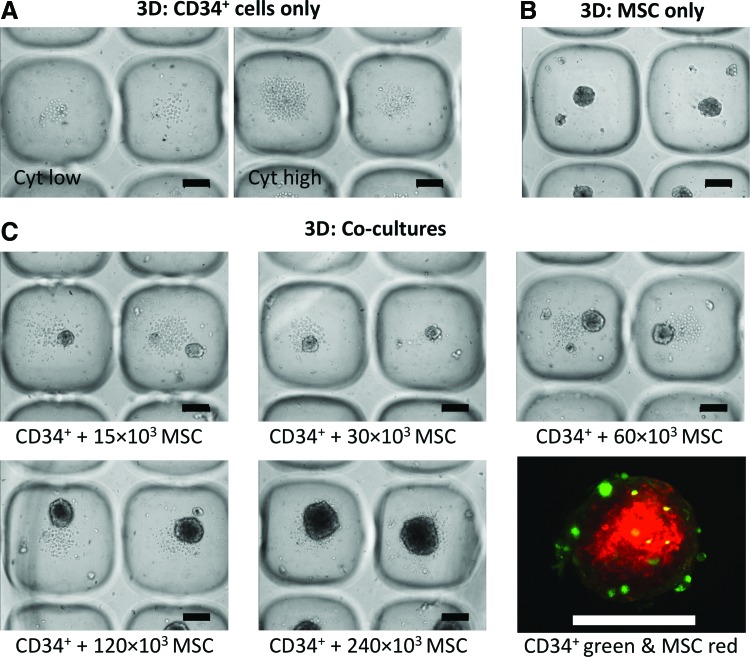
Phase-contrast images of 3D cultures after 7 days of expansion. **(A)** Cultures were prepared containing either 6 × 10^3^ CD34^+^ cells alone in low cytokine conditions (*left panel*) or high cytokine conditions (*right panel*). **(B)** 60 × 10^3^ MSCs only (100 MSCs per spheroid). **(C)** 6 × 10^3^ CD34^+^ cells (10 CD34^+^ per spheroid) and variable numbers of MSCs, as specified *below* images, in low cytokine conditions. *Bottom right* caption shows a confocal image of CFSE-labeled CD34^+^ cells (*green*) bound to CMTPX-labeled MSCs (*red*) at day 7 (these spheroids were formed from 10 CD34^+^ cells and 100 MSCs each). Scale bars = 100 μm. Color images available online at www.liebertpub.com/tec

Representative phase-contrast images of 2D and 3D cultures are shown in Figures 4 and 5, respectively. In both 2D cocultures and 3D cocultures, hematopoietic cells could be distinguished from MSCs as small bright/round cells, piled on top of the MSC monolayer, or surrounding the MSC spheroids, respectively. There was a visible increase in the number of hematopoietic cells in the 2D CD34^+^ cell-only cultures in response to medium supplementation with high concentrations of exogenous cytokines relative to low cytokine concentrations (Fig. 4A). In 2D cocultures seeded with variable numbers of MSCs, it was evident that adherent monolayers appeared denser with increasing MSC numbers (Fig. 4C). However, differences in the number of detached rounded cells (hematopoietic cells) were not visually apparent in response to increasing numbers of MSCs (Fig. 4C).

Similar to 2D cultures, there was a visible increase in the number of hematopoietic cells in CD34^+^ cell-only cultures in the 3D platform in medium that contained high concentrations of exogenous cytokines relative to low concentrations (Fig. 5A). MSC-only cultures formed and maintained their spheroidal geometry over the 7-day culture period (Fig. 5B). In 3D cocultures, not all CD34^+^ cells attached to the MSC spheroids (Fig. 5C). At 24 h, only ∼30% of CD34^+^ cells were physically anchored to the MSC spheroids (described previously^19^). At the day 7 harvest point, very few CD34^+^ (green) cells remained attached to the MSC spheroids (red) as shown in Figure 5C (bottom right panel). Most hematopoietic cells pooled in the microwells adjacent to, but not attached to, the MSC spheroids. This pooling at the base of spheroids was similar to results reported in two other recent spheroid coculture publications.^12,15^ The hematopoietic cells that were anchored to spheroids appeared to be localized to the outer surface of the spheroids, with MSCs forming the core. The number of hematopoietic cells was not uniform across all microwells at day 7, with some microwells being notably more confluent than others, and this pattern appeared random across the surface of the microwell array (Fig. 5A, C). This variable pattern of hematopoietic cell density across microwells is likely a reflection of the heterogeneity of the initial CD34^+^ cell populations,^38,39^ with individual cells having different proliferative potentials.

### Total cell expansion in 2D and 3D cocultures

**FIG. 6.**
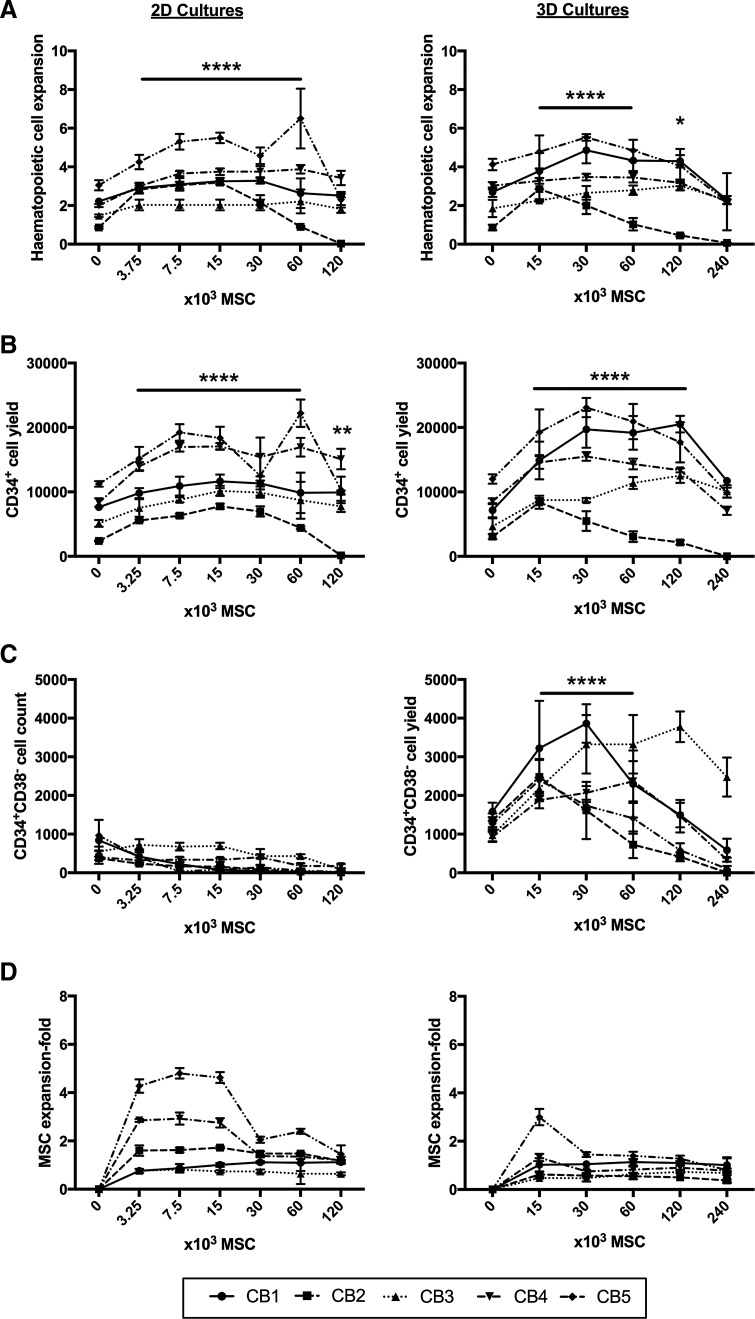
Comparison of 2D and 3D cocultures. Seven-day expansion cultures were initiated with 6 × 10^3^ CD34^+^ cells each (10 CD34^+^ cells per spheroid), and increasing numbers of MSCs, as indicated on the *x*-axis. **(A)** Total hematopoietic cell fold expansion. **(B)** Total CD34^+^ cell culture yield. **(C)** Total CD34^+^CD38^−^ cell culture yield. **(D)** Total MSC fold expansion. Five replicate data sets from independent experiments are shown in each caption. *Points* on the *lines* represent the mean ± SD for four replicate wells from a single study. Cultures from the same study are connected by a line. SD, standard deviation. Statistical significance for each comparison was performed using ANOVA, with significance indicators as described in the Methods and Materials/Statistical analyses section.

All 2D and 3D cultures were initiated with 6 × 10^3^ CD34^+^ cells per well, equating to ∼10 CD34^+^ cells per microwell in the case of 3D cultures. Using medium supplemented with low concentrations of exogenous cytokines (10 ng/mL SCF), we tested the capacity of different numbers of MSCs in both 2D and 3D cultures to support HSPC expansion (Fig. 6A–C). Experiments using a combination of three different MSC donors and five different CB donors (CB1-5) are represented as different line graphs in Figure 6A–C.

At day 7, cultures were digested into single-cell suspensions and characterized by flow cytometry to determine total hematopoietic cell expansion as well as CD34^+^ and CD34^+^CD38^−^ progenitor cell yield in 2D and 3D cocultures. Supplementary Figures S1 and S2 show flow cytometry scatter plots demonstrating how markers and gating were used to differentiate between MSC and hematopoietic cell populations.

The addition of MSCs in 2D and 3D cultures modestly improved total hematopoietic cell (CD45^+^ cells) expansion relative to zero MSC controls (Fig. 6A). The addition of 3.75 × 10^3^–60 × 10^3^ MSCs per well (or 1 cm^2^) was shown to statistically increase total hematopoietic cell expansion in 2D cocultures. In 3D, MSC support also improved total hematopoietic cell expansion when 15 × 10^3^–60 × 10^3^ MSCs were added per well in the cocultures; this equated to approximately 25–100 MSCs/microwell. On average, total hematopoietic cell expansion from the five replicate experiments (CB1–5 in Fig. 6) was greater in the presence of MSCs, but was statistically similar between the 2D and 3D MSC coculture platforms. In both 2D and 3D cocultures, supplementation of cultures with too many MSCs (>60 × 10^3^ MSCs per well) compromised total hematopoietic cell expansion, likely due to nutrient limitations at these high cell densities.

Each culture was initiated with a defined number of MSCs, as shown on the horizontal axis of the graphs in Figure 6. The number of MSCs at harvest was determined using flow cytometry, and MSC fold expansion plotted in Figure 6D. Modest MSC expansion was observed in 2D cocultures with CB donors 4 and 5 (CB4 and CB5), but only at lower MSC seeding densities (3.75 × 10^3^ to 15 × 10^3^ MSCs/well). Little to no MSC expansion was observed in the 3D cocultures, regardless of initial seeding density.

### CD34^+^ cell yield in 2D and 3D cocultures

Like total hematopoietic cell expansion, CD34^+^ cell yield at day 7 was greater in cultures that included MSC support cells (Fig. 6B) relative to cultures that contained zero MSCs. On average, a statistically greater CD34^+^ cell yield was observed when cultures were initiated with 3.75–60 × 10^3^ MSCs in 2D or 15–120 × 10^3^ MSCs (25–200 MSCs per spheroid) in 3D per well. When data across the five expansion experiments were averaged, CD34^+^ cell yield was statistically similar between 2D and 3D cultures. Like total hematopoietic cell expansion, CD34^+^ cell yield was compromised when too many MSCs (>60 × 10^3^ in 2D and >120 × 10^3^ in 3D) were added to cocultures.

### CD34^+^CD38^−^ cell yield in 2D and 3D cocultures

In 2D cultures, the addition of MSCs resulted in an incremental reduction in the yield of CD34^+^CD38^−^ cells in four of the five expansion cultures (Fig. 6C, left panel). The exception was donor CB3, where CD34^+^CD38^−^ yield was unchanged in response to MSC coculture support. The pattern of reduced CD34^+^CD38^−^ cell yield with increasing MSC numbers across the four experiments was statistically significant for MSC numbers >7.5 × 10^3^ per well in 2D.

A substantial increase in the CD34^+^CD38^−^ cell yield was observed in 3D cultures containing 15–60 × 10^3^ MSCs, relative to zero MSC controls, when averaged across the five CB expansion cultures (Fig. 6C, right panel). The maximal average CD34^+^CD38^−^ cell yield in these 3D cocultures was observed at 30 × 10^3^ MSCs per well (2.52 × 10^3^ ± 415 CD34^+^CD38^−^ cells), which was substantially greater than in 2D cocultures initiated with an equivalent number of MSCs (0.19 × 10^3^ ± 177 CD34^+^CD38^−^ cells). This represented an ∼13-fold increase in the yield of CD34^+^CD38^−^ cells in the 3D cocultures. The addition of >60 × 10^3^ MSCs per well in 3D cocultures was detrimental to overall CD34^+^CD38^−^ cell yield.

### CD38^−^ cell yield in 2D TCP and 3D microwell cultures

A consistently greater CD34^+^CD38^−^ cell yield was observed in the 3D cocultures (Fig. 6C). In the zero MSC control cultures (Fig. 6C), the average CD34^+^CD38^−^ cell yield across five CB donors in the 2D cultures was 0.62 × 10^3^ ± 304, while the CD34^+^CD38^−^ cell yield in the 3D microwell cultures was 1.22 × 10^3^ ± 297. These data indicated that the microwell culture platform yielded more CD34^+^CD38^−^ cells (*p* < 0.0001), even without MSCs.

**FIG. 7.**
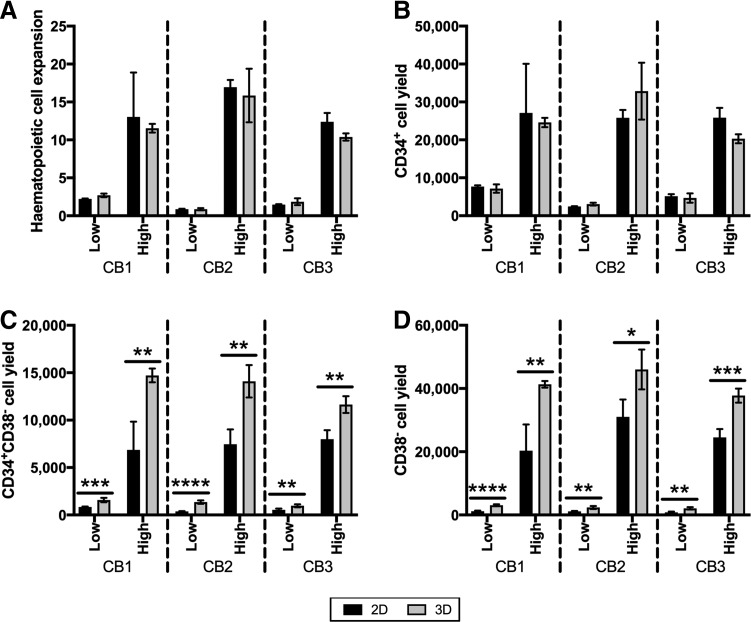
Characterization of cell yield in 2D and 3D cultures without MSCs to highlight effect of PDMS microwells on CD38. **(A)** Total cell expansion was similar in 2D and 3D cultures. **(B)** CD34^+^ cell yield was similar in 2D and 3D cultures. **(C)** CD38^−^ cell yield was statistically greater in 3D PDMS microwell cultures. **(D)** CD34^+^CD38^−^ cell yield was statistically greater in 3D PDMS microwell cultures. All cell number data were generated using flow cytometry, as described in the Materials and Methods section. Three replicate experiments are shown. For each experiment, bars represent the mean ± SD for four replicate wells. Statistical significance for each comparison was performed using *t*-test, with significance indicators as described in the Methods and Materials/Statistical analyses section.

To assess the impact of the 3D microwell platform on CD34^+^CD38^−^ cell yield, 2D and 3D microwell expansion cultures that were performed with low and high cytokine-supplemented media, but contained no MSCs, were evaluated (Fig. 7). Total hematopoietic (CD45^+^) cell expansion and CD34^+^ cell yield in cultures supplemented with low or high concentrations of cytokines were similar in 2D and 3D microwell cultures (Fig. 7A, B). By contrast, the yield of CD34^+^CD38^−^ cells and, more generally, CD38^−^ cells was significantly greater when cultures were performed in 3D microwells (Fig. 7C, D) in both low and high cytokine cultures.

### Does PDMS microwell culture modify engraftment potential?

**FIG. 8.**
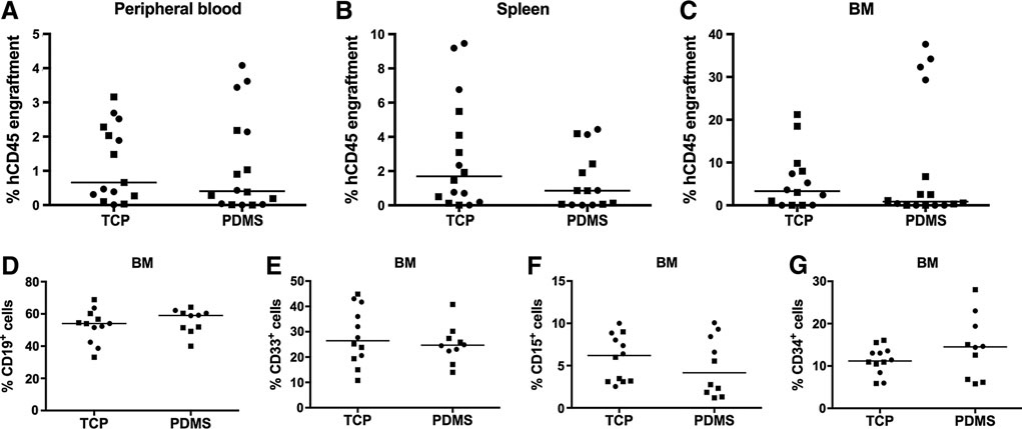
PDMS microwell culture-mediated CD38^−^ cell yield did not result in statistically significant differences in engraftment between groups. Two replicate experiments, using two different cord blood donors, are individually represented by *circle* or *square* symbols. Graphs show the percentage human engraftment (hCD45^+^) in NSG mice, detected in the **(A)** peripheral blood, **(B)** spleen, and **(C)** BM. Lineage composition of BM hCD45 cells, including **(D)** B cells (CD19^+^), **(E)** myeloid (CD33^+^), **(F)** granulocyte (CD15^+^), and **(G)** progenitor cells (CD34^+^). Individual mice represented as individual symbols (*circles* and *squares* represent two different cell donors). *Lines* are drawn at the median value. BM, bone marrow; NSG, NOD/SCID gamma.

As a major outcome from our studies was the increased yield of CD34^+^CD38^−^ cells resulting from culture in PDMS 3D microwell cultures, we quantified the relative capacity of CD34^+^ cells expanded on TCP versus PDMS microwells to engraft in NSG mice. For these studies, we transplanted in mice CD34^+^ cells that were expanded in high cytokine conditions for 7 days on either TCP or in PDMS microwells. When transplanted into NSG mice, the greater yield of CD34^+^CD38^−^ cells derived in the PDMS microwell cultures did not confer an engraftment advantage. The percentage of human cells in the peripheral blood, spleen, or BM of NSG mice was similar regardless of prior expansion on TCP or in PDMS microwells (Fig. 8A–C). Similarly, the greater proportion of CD34^+^CD38^−^ cells derived from PDMS cultures did not modify the lineage potential of engrafted cells (Fig. 8D–G).

## Discussion

Contemporary stromal cell coculture systems are established by seeding HSCs onto 2D stromal cell monolayers. Clinical studies have demonstrated that HSCs expanded in such cultures do not engraft long term in human recipients,^4,5^ suggesting that these culture conditions deplete expanded cell populations of long-term engrafting HSCs, and likely only contain lineage-committed progenitor cells. Previous literature reports have suggested that the assembly of MSCs into spheroids increased their HSPC-supportive gene expression and secretion profiles,^31,37,40–42^ as well as that HSPCs could be maintained when cocultured *in vitro* with MSC spheroids.^12,15,16,43^ Our study assessed HSPC expansion outcomes in a microwell platform that facilitated the production of hundreds of MSC spheroids, each containing precise numbers of MSCs, in coculture.

### MSC 2D and 3D gene expression analysis using microarrays

Previous studies using gene microarrays reported that gene expression profiles were changed when MSCs were cultured as 3D spheroids relative to 2D adherent monolayers.^31,37^ In these previous studies, the hanging drop technique was used to produce relatively large MSC spheroids (formed from 25 to 250 × 10^3^ cells per spheroid), which were cultured in serum-containing medium in a 20% O_2_ atmosphere. The spheroids evaluated in our gene microarray study were formed from ∼100 MSCs each, assembled using a high-throughput microwell array system, and compared with 2D monolayer cultures. Cells were cultured in commercial serum-free medium in a 2% O_2_ atmosphere. We identified more than 140 genes that were significantly upregulated more than twofold and more than 200 that were downregulated more than twofold at days 3 and 6 of culture (Table 1).

Osteopontin (SPP1 or OPN) was the most differentially upregulated gene on day 3 and second most upregulated gene on day 6 in 3D cultures compared with 2D cultures. OPN is a secreted protein involved in modulating biomineralization, cell adhesion, antiapoptosis, and immune modulation (reviewed in Sodek *et al.*^44^). OPN has been shown to suppress murine HSC expansion *in vitro* and OPN knockout mice have been shown to exhibit a marked increase in HSC cycling.^45^ OPN cleavage fragments have been shown to bind integrins expressed on HSCs, resulting in their attraction, retention, and regulation in the BM niche.^46^ Insulin-like growth factor-binding protein-1 (IGFBP1) was upregulated in 3D MSC spheroid cultures and has previously been shown to improve CD34^+^ progenitor cell expansion when used in combination with other cytokines.^47^ Secreted factors known to induce osteogenesis in osteoprogenitor cells were also among the most highly differentially expressed genes in 3D, including bone morphogenetic protein (BMP)-2, BMP-6, and TGF-β2.^48–50^ These factors have also been reported to influence HSPCs^51,52^; however, their influence has been rather less definitively delineated and response depends on the intrinsic HSPC state, such as age or lineage bias.

Other groups have previously compared differentially expressed genes of MSCs cultured in 2D monolayer and 3D spheroid cultures (Potapova *et al.*^37^ and Bartosh *et al.*^31^). Based on the comparison in Table 2, it was apparent that upregulation of some genes was common between previous studies and our current study (e.g., BMP-2, SPP1/OPN, APOD, and NR4A2). However, the majority of values reported by Potapova *et al.*^37^ and the current study (at both days 3 and 6) did not always appear to have the same magnitude of upregulation as reported by Bartosh *et al.*^31^

Differentially expressed genes in our study were over-represented in functional gene ontologies related to the extracellular region, extracellular matrix, response to oxygen and hypoxia, regulation of cellular and biological processes, and bone mineralization (Fig. 3). As in our study, Potapova *et al.*^37^ assessed a single MSC donor, while Bartosh *et al.*^31^ assessed two MSC donors. The source of variation in gene expression between studies may reflect donor variability and/or the different culturing techniques used in each study. A recent follow-up study reported that when MSC spheroids were cultured in serum-free medium, they did not possess the same anti-inflammatory gene expression pattern and functional properties observed when MSC spheroids were cultured in serum-containing media.^42^ This is consistent with the results observed in our study, where we did not see significant changes in anti-inflammatory gene expression. Unlike the Potapova *et al.*^37^ and Bartosh *et al.*^31^ studies, we used serum-free medium designed for hematopoietic cell expansion as serum is known to contain factors that promote HSPC differentiation.^53^ As such, the differences in gene expression patterns are likely strongly influenced by our selection of serum-free medium.

Factors that have been characterized as key HSPC-supportive cytokines, such as SCF, TPO, Flt-3 ligand, CXCL12, and Notch ligands,^1^ were not found to be significantly upregulated in our 3D MSC spheroid cultures compared with 2D. This observation differs from previous studies using MSC spheroids enriched for subpopulations (PDGFRα^+^ and CD51^+^ cells^12^ or CD146^+^ cells^13^), which reported the upregulation of genes characteristic of HSPC support. These authors used enriched MSC subpopulations and different base media, both of which could have contributed to their observed upregulation of specific HSPC-supportive factors in 3D spheroids. It is also noteworthy that our and previous gene expression analyses were performed on MSCs cultured as spheroids in isolation, rather than in coculture with HSPCs. Cross-talk between MSCs and HSPCs has been shown previously,^54,55^ demonstrating that there are some MSC gene expression changes in response to factors expressed by hematopoietic cells. Thus, it is possible that HSPC-supportive gene expression by MSCs might be altered when these cells are cultured in the presence of HSPCs. In our studies and previous studies mentioned above, a range of gene expression differences between the 2D and 3D spheroid cultured cells were observed. However, there remains a knowledge gap on precise MSC-expressed genes that might be responsible for conferring HSPC support *in vitro*, making gene expression analysis on its own insufficient to optimize support cell cultures.

In our coculture studies, the MSC donor cells and CB donor cells were not from the same individual. Using this approach in a clinical scenario would mean that both the MSC and CB cell populations would be allogeneic relative to the CB transplant recipient. This approach is common in the literature and reflects both the ethical/logistical challenge of obtaining a BM aspirate from the CB donor and the pressure to develop an affordable therapy. A key economic advantage of this approach is that MSCs isolated from a single donor could be expanded and characterized and used as an off-the-shelf product to support coculture of multiple units of CB.^56^ While this has not been explored in great detail, evidence does suggest that third-party MSCs (MSCs not from the CB donor or the CB transplant recipient) can be used in cocultures without compromising clinical outcomes.^57^ The use of autologous cells from the CB transplant recipient introduces the risk of increased MSC donor variability, cost, and treatment delay. Moreover, some studies have reported that MSCs derived from diseased donors can have slower division rates, contain abnormalities, and may alter the behavior of healthy hematopoietic cells, compared with healthy controls.^58,59^

Extensively expanded third-party MSCs have been applied most widely in human CB-derived HSPC expansion studies.^5,57^ However, the most promising HSPC expansion cocultures described in preclinical studies have used enriched subpopulations of MSCs with minimal *in vitro* expansion.^12,15^ While *in vitro* expansion of MSCs is known to alter their HSPC-supportive capacity,^60^ considerable expansion would be required to generate multiple units of an off-the-shelf cell product.^56^ Thus, there will likely be a sweet spot, at which MSC expansion delivers sufficient cell numbers to support coculture of multiple units of CB, but which does not excessively compromise their biological characteristics. These expanded third-party MSCs may potentially also be useful in facilitating engraftment when cotransplanted with HSPCs^61^ and/or delivered at a later time point to treat graft-versus-host disease (GVHD).^62^ All of these applications could potentially be improved if the biological potency of MSCs was truly increased when these cells are assembled into 3D spheroids.

### Two-dimensional and 3D coculture characterization

In our 3D spheroid system, the majority of hematopoietic cells pooled in microwells at the base of the spheroid (Fig. 5). When hematopoietic cells did attach to MSC spheroids, they were largely localized to the outer surface, as shown previously.^19^ We also observed similar 3D organization and pooling of hematopoietic cells around MSC spheroids in an earlier study using murine Lin^−^Sca^+^Kit^+^ (LSK) hematopoietic cells combined with murine BM-derived MSCs.^17^ Previous studies that assessed the organization of two different cell types in a common spheroid demonstrated that the cell population with the greatest level of adherence to its own cell type tended to form the core of a spheroid, while the cell population with the lowest level of adherence to its own cell type formed an outer shell.^63^ Consistent with this study, we observed that the nonadherent hematopoietic cells were located on the outside of spheroids, while MSCs formed the core. While many hematopoietic cells did not remain attached to the MSC spheroids, the microwells promoted pooling of detached hematopoietic cells in close proximity to MSC spheroids.

It is unclear if direct hematopoietic cell contact with the MSC spheroid is essential to achieve a positive culture outcome. Corselli *et al.* identified CD146^+^ perivascular cells as a key HSPC-supportive fraction and reported that direct contact was required for these MSCs to support HSPCs through notch signaling.^13^ Pinho *et al.* identified PDGFRα and CD51 as markers of an HSPC-supportive MSC subpopulation and reported that spheroids formed from these cells were capable of supporting HSPCs through indirect soluble signaling.^12^ Similarly, Isern *et al.* found that when the CD146^+^/nestin^+^ fraction of human MSCs was isolated and cultured as spheroids, they supported HSPCs *in vitro* through indirect soluble signaling.^15^ It is possible that the close proximity of HSPCs to MSC spheroids, in the microwells, might be sufficient to enhance HSPC coculture outcomes and direct contact may not be necessary.

### Total cell expansion in 2D and 3D cocultures

In both 2D and 3D cultures, a parabolic response in total hematopoietic (CD45^+^) cell expansion was observed, whereby the addition of MSCs at lower numbers improved hematopoietic expansion until a maximum was reached, at which the addition of more MSCs compromised expansion outcomes. When data points were averaged from the five CB expansion experiments, there was no advantage in total hematopoietic (CD45^+^) cell expansion associated with 3D cocultures over 2D cocultures.

Greater MSC expansion was observed in 2D cocultures than in 3D cocultures, although MSC expansion was modest in both cases. Limited expansion of MSCs in 2D and 3D cocultures was expected as the culture medium was serum free, 2D monolayers were seeded near confluence in the higher MSC-containing cultures, and 3D MSC spheroids cultured in similar platforms have been previously reported to undergo minimal proliferation and compaction in size over similar culture durations.^35^ Neither the 2D system nor the 3D system used here is optimal for extensive MSC expansion, and MSC expansion is not known to be necessary for *ex vivo* HSPC support.

### CD34^+^ cell yield in 2D and 3D cocultures

The surface expression of CD34 on hematopoietic cells is widely used as a marker of progenitor cell-enriched populations.^3–6^ In low cytokine-supplemented media, CD34^+^ cell yield was only modestly improved with the addition of MSCs in 2D and 3D cultures. Given that cultures were initiated with 6 × 10^3^ CD34^+^ cells per well, the maximal yields (∼20 × 10^3^ CD34^+^ cells) represented an approximately three- to fourfold expansion of the input CD34^+^ cells. Like total hematopoietic cell expansion, there was a parabolic CD34^+^ cell yield response to MSCs with the addition of lower numbers of MSCs improving CD34^+^ cell yield until a maximum point was reached, after which adding more MSCs reduced CD34^+^ progenitor cell yield both in 2D and 3D cultures. This observation is consistent with Isern *et al.* who reported that the addition of too many MSC spheroids to cocultures compromised HSPC cell yield.^15^ When data points from all five CB expansion cultures were averaged, there was no statistical advantage associated with the 3D culture platform compared with 2D.

Our CD34^+^ cell yields in MSC spheroid coculture were more modest than previously reported by others.^12,15^ Isern *et al.* observed approximately 6- or ∼40-fold increase in CD34^+^ cells depending on whether culture medium was supplemented with human serum or chick embryo extract, respectively.^15^ Pinho *et al.* also observed more substantial expansion of HSPCs, but their minimal medium contained more cytokines (25 ng/mL SCF, 12.5 ng/mL TPO, and 25 ng/mL Flt-3 ligand).^12^ Recently, Corselli *et al.* reported only a delay in the depletion of HSPCs in 2D cocultures that were initiated with CD146^+^-enriched support MSCs and supplemented with medium containing 5% FBS, but no exogenous cytokines.^13^ It appears that the exogenous factors contained either in serum products, chick embryo extract, or the addition of various types of cytokines may augment MSC secretions and support HSPC expansion variably.^12,15^

Our overall goal was to develop a bioprocess that might produce a cell product suitable for clinical use. While it is common to use cytokine and growth factor concentrations ranging from 10 to 100 ng/mL for *in vitro* cultures, the physiological concentrations of most factors in the BM are in the picogram/mL range.^64^ HSPCs expanded in MSC cocultures using combinations of SCF, TPO, and/or Flt-3 ligand at higher concentrations (∼100 ng/mL) have been trialed clinically, but have thus far not yielded cell populations capable of long-term engraftment in human recipients.^1,5^ More broadly, highly cytokine-supplemented media that yielded large numbers of hematopoietic cells have failed to produce cell products that engraft long term in human recipients.^1,3,4^ To this end, we used a serum-free, FDA-approved base medium (X Vivo-15) and avoided undefined animal products such as FBS and chick embryo extract. Furthermore, to elucidate the capacity of MSCs to support HSPCs in 2D and 3D cocultures, we supplemented our media with minimal HSPC-supportive cytokine (10 ng/mL of SCF).

In our 3D cocultures, the CD34^+^ cell population increased approximately three- to fourfold following 7 days of expansion. A fourfold increase could have therapeutic benefit if the expanded cells were functionally equivalent to cells present in unmanipulated CB as this would be equivalent to transplanting 4 U of CB instead of one. However, the modest total hematopoietic cell expansion would likely be insufficient to provide significant short-term myeloid support.^1^ Previous clinical studies that did observe measurable short-term myeloid support did so with CB CD34^+^ expansion ranging from 40- to 100-fold.^3–5^ To be of value, our observed modest expansion would have to yield cells with potent long-term engraftment potential. To better understand the phenotype, and predict engraftment potential of the expanded cell population, we quantified the CD34^+^CD38^−^ cell yield in 2D and 3D cocultures.

### CD34^+^CD38^−^ cell yield in 2D and 3D cocultures

CD34^+^CD38^−^ is considered to be a reliable marker of hematopoietic cells enriched for long-term engraftment capacity in immunocompromised murine models^65^ and is still commonly reported in CB expansion studies.^66,67^ The yield of CD34^+^CD38^−^ cells in 2D and 3D expansion cultures is shown in Figure 6C. In 2D cultures, the incremental addition of more MSCs, across four of five CB expansion cultures, resulted in an incremental reduction in the CD34^+^CD38^−^ cell yield. Unlike 2D cultures, the yield of CD34^+^CD38^−^ cells in 3D cultures increased significantly in response to the addition of MSCs, across the five CB expansion cultures. A maximal CD34^+^CD38^−^ cell yield in 3D cocultures, relative to no MSC controls, was observed when spheroids were formed from 50 MSCs each (or 50 × 10^3^ MSCs per well). Relative to zero MSC 3D control cultures, this condition had an ∼13-fold higher CD34^+^CD38^−^ cell yield. This promising result suggested that our 3D cultures might yield a larger population of cells with long-term engraftment capacity.^65^

### CD38^−^ cell yield in 2D and 3D microwell cultures

A striking feature of the 3D microwell cultures was the consistently greater CD34^+^CD38^−^ cell yield relative to equivalent 2D cultures (Fig. 6C). However, the pattern of greater CD34^+^CD38^−^ cell yield in 3D cultures also occurred in the absence of MSCs (see zero MSC controls in Fig. 6C). Data from 2D and 3D cultures that did not contain MSCs, but were maintained in low and high cytokine-supplemented medium, are shown together in Figure 7. This analysis demonstrated that total hematopoietic cell expansion and CD34^+^ cell yield did not differ when the hematopoietic cells were cultured on 2D surfaces or on the 3D microwells (Fig. 7A, B). However, the CD34^+^CD38^−^ cell yield and overall CD38^−^ cell yield were significantly greater when cultures were performed in 3D PDMS microwells (Fig. 7C, D).

The microwell platform used in our 3D expansion cultures was made from PDMS polymer, which is considered to be noncytotoxic and relatively inert.^68^ However, our work here indicated that CD38 cell surface expression was significantly reduced in PDMS microwells. We have since published work showing that PDMS can absorb all-*trans* retinoic acid (ATRA) in a time-dependent manner, thereby depleting it from the cell culture medium.^23^ CD38 is a cyclic ADP ribose hydrolase and its expression can be induced by ATRA.^69^ Thus, depletion of ATRA from the cell culture medium by the PDMS could be a potential mechanism causing the increase in CD38^−^ cell yield observed in microwell cultures. The commercial serum-free medium (X Vivo-15) used in our studies was not supplemented with exogenous ATRA, and we were unable to detect ATRA in this medium using fast LC-multistage tandem mass spectrometry.^23^ X Vivo-15 does contain human serum albumin, which is known to carry trace quantities of retinoids.^70,71^ In our previous work, we showed that CB cells do not have to be cultured in the presence of PDMS, rather preincubation of X Vivo-15 medium on PDMS is sufficient to augment subsequent CD38 gene and cell surface protein expression.^23^ Similarly, culture of CB cells on flat surfaces of PDMS reduced CD38 surface expression, suggesting that material absorption of some factor by the PDMS indirectly contributes to the change in CD38 expression. Retinoids are notoriously challenging to quantify,^72^ and it is probable that this medium contains some retinoids at concentrations below our detection limits. Whether depletion of trace quantities of ATRA from medium by PDMS is sufficient to modify CD38 expression or whether other factors in the media may be augmented by PDMS remains unknown.

### Does PDMS microwell culture modify engraftment potential?

We evaluated the relative engraftment capacity of CD34^+^ cells expanded on either TCP or PDMS using an NSG mouse transplantation assay. We reasoned that improving the cell product without the complexity, variability, and cost of the MSC coculture would be a major achievement. Unfortunately, the greater CD34^+^CD38^−^ cell yields derived in the PDMS cultures did not confer an engraftment advantage in the peripheral blood, spleen, or BM of the recipient mice (Fig. 8A–C). The lineage potential of the engrafted cells was also unmodified by PDMS culture (Fig. 8D–G). Our results are perhaps not surprising as others have reported that there is a disconnect between CD38 cell surface expression and functional phenotype following *ex vivo* culture.^65,73–75^ Specifically, previous reports have noted that CD38 cell surface expression drops rapidly during culture in serum-free medium, but that the associated increase in CD34^+^CD38^−^ yield does not confer an increase in engraftment capacity in immunocompromised mice.^73,74^ In our 3D system, PDMS likely exacerbates the rapid depletion of a hydrophobic CD38-inducing factor from the culture medium.

Cumulatively, our data further support that CD38 is not a reliable marker of the engraftment potential of cells expanded *in vitro*, particularly in the presence of hydrophobic materials such as PDMS. In our study, the CD34^+^ cell yield was a better predictor of the similar engraftment potential of 2D and 3D microwell expansion cultures. Recently, human HSC surface markers have been identified, including CD90, CD45RA,^76^ and CD49f,^7^ as well biophysical^77^ and biochemical^7,78^ assays, which when used in combination with CD34, facilitate the characterization of engraftment potential in NSG mice. These markers should be used in preference to CD38 for evaluating expanded HSPC cultures.

### Summary

We reasoned that HSPC coculture outcomes could be improved if the supportive MSCs were present in the form of 3D spheroids. The rationale behind these experiments was driven by our efforts to generate a more 3D-organized culture mimic and literature suggesting that spheroid cultures could be used to modulate MSC gene expression and secretion profiles,^31,37,40–42^ as well as reports suggesting that HSPCs could be maintained *in vitro* using MSC spheroids.^12,15,16,43^

Using microarrays, we evaluated differentially expressed genes when MSCs were cultured as 3D spheroids in a microwell platform relative to 2D monolayers. While there were differences in gene expression patterns, the gene expression of well-known HSC maintenance factors such as SCF, TPO, or Notch ligands,^1^ for instance, did not differ between 3D and 2D MSC culture in our study.

CD34^+^ cell expansion was evaluated when cocultured with MSCs in a 3D spheroid microwell platform and compared with that of traditional 2D monolayer cocultures. HSPC expansion improved modestly with MSC support cells with minimal cytokine supplementation in both 2D and 3D cultures. There may be a number of opportunities to improve on this platform, including the use of more enriched MSC populations,^12,13^ and possibly the optimization of medium supplements.

Coculture with MSCs in 2D generally resulted in depletion of CD34^+^CD38^−^ progenitor cells. By contrast, cultures maintained in PDMS microwells yielded a greater number of CD34^+^CD38^−^ cells. The increase in yield of CD34^+^CD38^−^ cells was shown to occur in response to the presence of the PDMS microwell insert and was independent of MSCs. The increase in CD34^+^CD38^−^ cell yield generated in the PDMS expanded cultures did not confer a detectable increase in human hematopoietic cell engraftment in NSG mice. While PDMS microwell culture increases the yield of CD34^+^CD38^−^ cells, this culture approach did not appear to modify the engraftment potential of the cell product, further supporting the notion that CD38 is a poor marker of engraftment potential in expanded CB cells.

## Supplementary Material

Supplemental data

Supplemental data

Supplemental data
