# Supplementary material for: Spheroid Coculture of Hematopoietic Stem/Progenitor Cells and Monolayer Expanded Mesenchymal Stem/Stromal Cells in Polydimethylsiloxane Microwells Modestly Improves In Vitro Hematopoietic Stem/Progenitor Cell Expansion
Source: Tissue Eng Part C Methods. 2017 Apr 1;23(4):200–18. doi: 10.1089/ten.tec.2016.0329 (PMC5397247; doi:10.1089/ten.tec.2016.0329)
Supplement: Supplemental data [file Supp_Fig1.pdf]

Supplementary Data

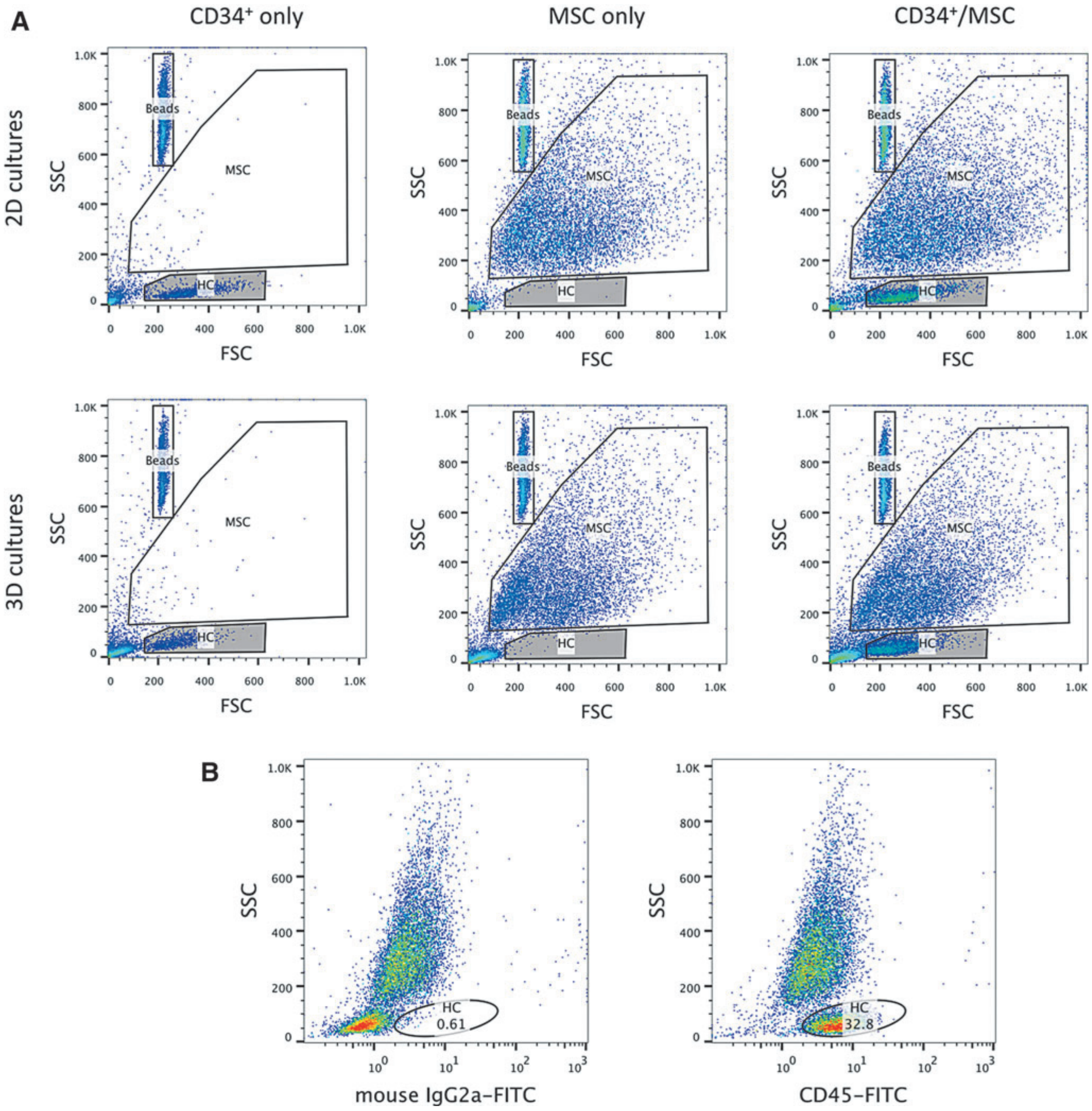

SUPPLEMENTARY FIG. S1. Hematopoietic cell (HC) and MSC discrimination based on FSC and SSC and CD45 expression by flow cytometry. FSC SSC plots are shown for expanded cells initiated from cultures containing $CD34^+$ cell-only (*left panels*), MSC-only (*middle panels*), and $CD34^+$/MSC cocultures (*right panels*) for 2D (*top panels*) and 3D (*bottom panels*) (**A**). CD45 expression was confirmed to be present on the hematopoietic cell population and not MSC population, as shown (**B**). Note: populations (**B**) are gated to include both HC and MSCs. The *left panel* shows cocultured cells stained with FITC-conjugated isotype control and the *right panel* shows cocultured cells stained with anti-human CD45-FITC-conjugated antibody. 2D, two-dimensional; 3D, three-dimensional; MSCs, mesenchymal stem/stromal cells.
