# Supplementary material for: Spheroid Coculture of Hematopoietic Stem/Progenitor Cells and Monolayer Expanded Mesenchymal Stem/Stromal Cells in Polydimethylsiloxane Microwells Modestly Improves In Vitro Hematopoietic Stem/Progenitor Cell Expansion
Source: Tissue Eng Part C Methods. 2017 Apr 1;23(4):200–18. doi: 10.1089/ten.tec.2016.0329 (PMC5397247; doi:10.1089/ten.tec.2016.0329)
Supplement: Supplemental data [file Supp_Fig2.pdf]

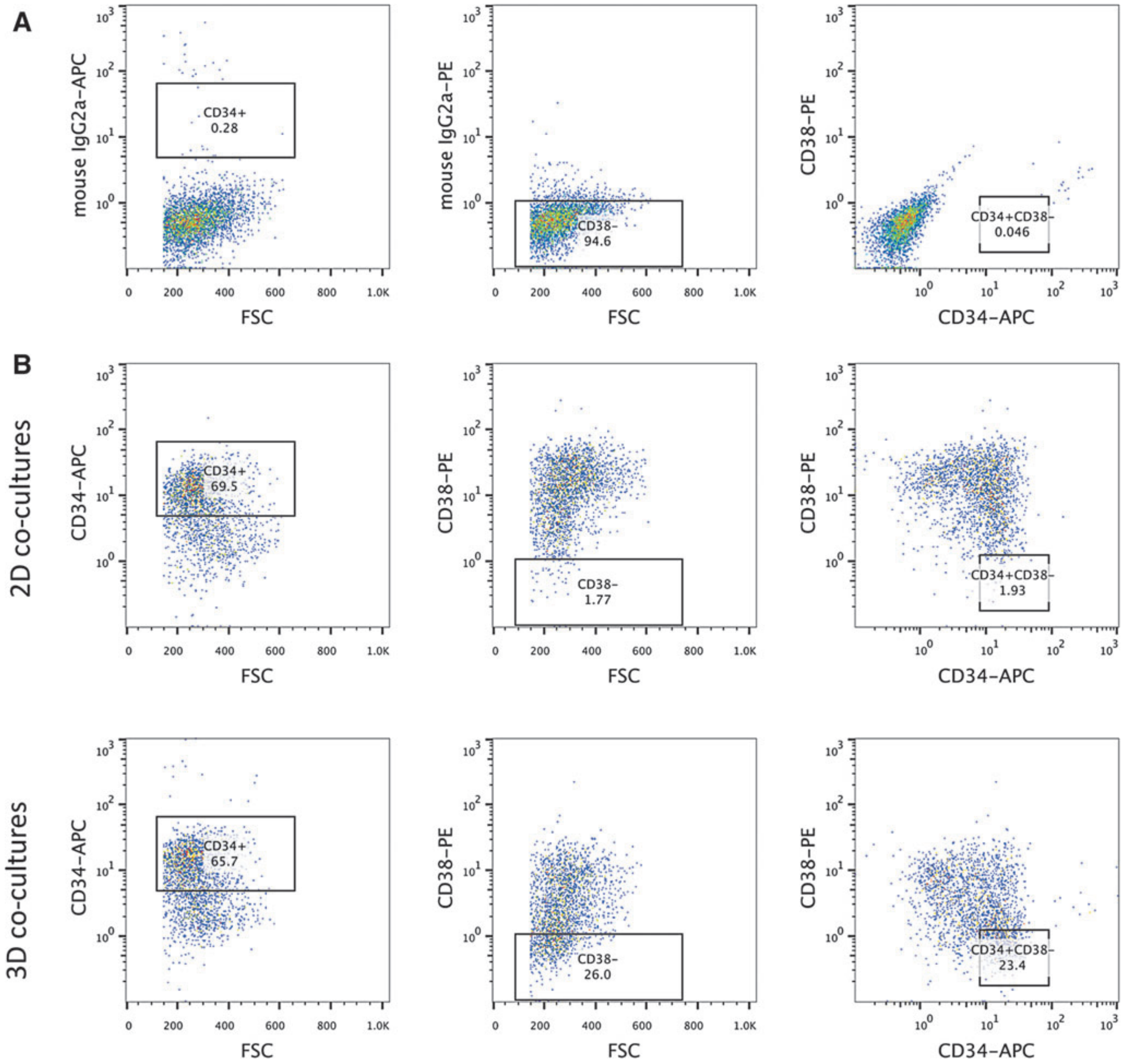

SUPPLEMENTARY FIG. S2. Flow cytometry gating strategy for hematopoietic progenitor cell surface marker expression on expanded $CD34^+$ cells. Cells stained with isotype controls for CD34-APC (*left panel*) and CD38-PE (*middle panel*) **(A)**; and single color-stained sample with anti-CD45-FITC, shown in the APC and PE channel (*right panel*) **(A)**. Expanded hematopoietic cells were first gated on FSC SSC, followed by $CD45^+$ (see Supplementary Fig. S2), and then subsequently by gating for $CD34^+$ cells, $CD38^-$ cells, and $CD34^+CD38^-$ cells **(B)**. Representative fluorescence plots for 2D cocultures are shown in *top panels* and for 3D cocultures in *bottom panels*.
